# High-speed railway infrastructure leads to species-specific changes and biotic homogenisation in surrounding bird community

**DOI:** 10.1371/journal.pone.0301899

**Published:** 2024-04-10

**Authors:** Lourenço Falcão Rodrigues, Cristina Mata Estacio, Jesús Herranz Barrera, Ana Eugenia Santamaría Figueroa, Juan Esteban Malo Arrázola

**Affiliations:** 1 Terrestrial Ecology Group, Department of Ecology, Autonomous University of Madrid (UAM), Madrid, Spain; 2 Centro de Investigación en Biodiversidad y Cambio Global (CIBC-UAM), Madrid, Spain; Universidad de Sevilla Facultad de Ciencias Económicas y Empresariales: Universidad de Sevilla Facultad de Ciencias Economicas y Empresariales, SPAIN

## Abstract

Linear infrastructure networks, including railways, are undergoing rapid development in order to connect distant urban areas. Particularly, High-Speed Railways are increasingly seen as a viable alternative to domestic flights in many countries. However, this development of linear infrastructures is known to affect the surrounding faunal communities due to the changes in the landscape and operation of said linear infrastructures. Both positive and negative effects of linear infrastructures on adjacent faunal communities have been reported. In this study, we determined the influence of the High-Speed Railway infrastructure on the bird community that surrounds it. Birds were surveyed by using both linear transect and direct counting methods, both in the area directly adjacent to the railway infrastructure and 500m away from it in a period of two years of surveys. A total of 16114 individuals belonging to 71 species were recorded. The presence of the High-Speed Railway caused species-specific changes in the bird communities that surround it, causing the attraction of some species and the rejection of others. Furthermore, we show that the presence of the infrastructure altered the natural species turnover as the landscape changes by attracting the same bird species regardless of changes in the landscape, and filtering out others. We propose that further work in mitigation and development plans should focus on species-specific measures to assess the risk bird communities are exposed to.

## Introduction

Human activities are widely known to affect the environment surrounding them, which leads to extensive research being done and targeted policies being put in place in order to assess, prevent and mitigate these effects on the environment [1–3]. Humans increasingly tend to concentrate in small but growing urban areas separated by extensive land masses. In order to connect these urban centers, linear infrastructure networks in general, and railway lines in particular have suffered rapid development in the last decades, and are expected to keep doing so in the future [4–7]. Specifically, high-speed railways (HSRs) are fast becoming a viable alternative to domestic flights in many countries, reducing the environmental impact of travel [5, 8].

The development of linear infrastructures, like HSRs, has become one of the main causes of landscape change [9, 10]. Structures associated with linear infrastructures are among the most notable elements of the anthropogenic landscape [10–12], and they extend for thousands of kilometres connecting urban centers. The construction and operation of linear infrastructures like roads and railways, produces several impacts in the ecosystems near the infrastructure. In the case of railways, very little is known about the effects they cause [13, 14]. Most knowledge in this regard has been extrapolated from the much better known field of road ecology, or included as a secondary part of these studies (e.g. [10, 15–17], see [14]). However, there are key differences between railways and roads that highlight the importance of the independent research of the impacts caused by railways [18]. These differences are found both in the infrastructure itself (presence of catenary and other accesory structures in railways) and in the nature of the traffic (intermittent in railways, and much higher speed in HSRs) [10, 18]. The evidence currently available for railways shows that the ecological effects of railways on surrounding wildlife communities are generally negative [18–20], although evidence of positive effects has also been noted [10, 21, 22]. Thus, the final effect of railways on the communities around them could be a balance of negative and positive effects, with the railways acting as species filter that attracts synanthrope species and repels species that reject anthropogenic infrastructures. These balanced effects are known to cause biotic homogenisation in urban centers [23–25]. However, very little is known on how linear infrastructures such as railways contribute towards biotic homogenisation.

Negative effects of railways include habitat clearing to make way for the infrastructure, and degradation of the remaining surrounding habitat during operation [18, 20]. This transformation of the habitat, and the presence and operation of railways poses an important threat to biodiversity [1]. Railway lines, just like roads, contribute towards habitat fragmentation, as the linear infrastructure divides the remaining available habitat into smaller patches [10, 14, 26, 27]. This affects the distribution of fauna, by conditioning its movement patterns [27, 28]. Furthermore, the railway infrastructure represents a barrier, which undermines connectivity among the patches of land that the rail divides [14]. Although railways may represent a larger physical barrier for non flying animals [27], they may also represent a behavioural barrier for some flying species too [14]. In the case of bird communities, direct mortality due to collisions with trains is of particular concern. For gregarious species, risk of collision is larger, as they tend to move in big flocks, making them vulnerable to collisions with linear infrastructure elements [29] or trains [30]. Disturbances like noise, lights and vibrations caused by passing trains also cause impacts to animals in the adjacent areas [14, 20, 31], which may preclude the presence of certain species, and has been found to inhibit the reproduction of certain species of birds [10, 32, 33].

However, positive effects of linear infrastructures, including railways, have been reported [10]. Linear infrastructures can increase biodiversity through edge effects as they run along different habitats [34]. The infrastructure associated with transport corridors creates new niches by providing new sources of food (small mammals, insects, grains…etc.) and perching and nesting places [10, 21, 35, 36]. Thus, birds of prey have been found to hunt in the areas close to the transport lines [35], and other birds have been found to nest on the verges of the transport lines, and even on the infrastructure associated with it [10, 37–39]. However, these attraction effects that railways may have on birds may be a double-edged sword. Even if biodiversity or number of observed individuals is increased by the presence of transport infrastructure, the species that are attracted to the infrastructure may be more prone to collisions with trains or the electrical infrastructure, in the case of railways. In the case of nesting behaviour, edge effects caused by the transport lines may increase the presence of predators, reducing the nestling survival rate and thus reproductive success [40, 41].

The main objective of our study was to determine the effect of the presence of the HSR infrastructure on the bird community in the area that surrounds it. In order to understand this, we explored the following questions: 1.- Whether the presence of the HSR infrastructure has an effect in the general abundance of birds, by either reducing or increasing the number of individuals; 2.- Whether the effect is mixed and species specific, attracting determined species to the rail and causing rejection in others; 3.- Whether the HSR has an homogenising effect on the species composition at the landscape scale, by comparing two different areas separated by several kilometers. Our results assess community level and species-specific effects of the presence of HSR infrastructure that will be invaluable in informing prevention and mitigation measures for the future development of new HSR lines.

## Materials and methods

### Data collection

#### Study area

**Fig 1:**
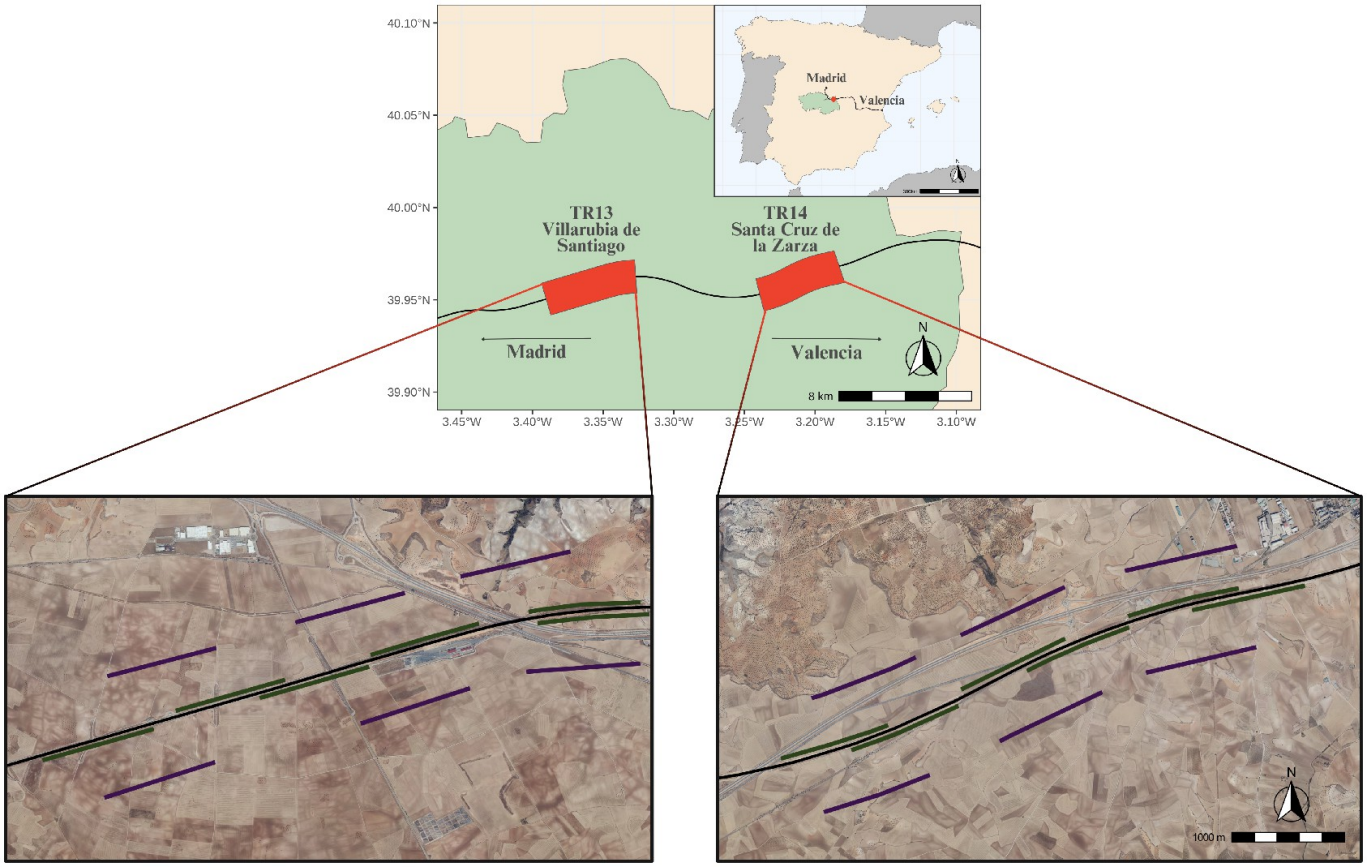
Map of the study area, represented in a section of the province of Toledo, central Spain. Green area delimits the Province of Toledo. The red dot in the map of Spain represents the location of the study site, and the red areas in the general view of the study site describe the two census areas (TR13-Villarrubia de Santiago and TR14-Santa Cruz de la Zarza). The picture images represent the two distinct census areas in higher detail. The pink lines in the images represent the transects by the rail and the blue lines represent transects 500m from the rail. Note that, for schematic purposes and visibility in the figure, transects by the rail were drawn slightly separated from the railway line, but they were performed very close to it, in reality. The black line crossing the census areas and going across the general map of Spain represents the High-Speed Railway Madrid—Castilla La Mancha—Comunidad Valenciana line. Reprinted from ign.es under a CC BY 4.0 license, with permission from IGN (Spanish National Geographic Institute), original copyright PNOAHISTORICO 2004–2019, scne.es.

We studied bird abundance in areas surrounding a HSR in a section of the Madrid—Castilla La Mancha—Comunidad Valenciana—Región de Murcia high-speed railway line situated in the province of Toledo, central Spain. Specifically, the study area was situated in the Ocaña-Villarrubia de Santiago and Villarrubia de Santiago-Tarancón subsections of the high-speed railway line (Fig 1). The total length of the study site amounts to 10 645m and it is subdivided in two distinct census areas: TR13-Villarrubia de Santiago, with 5 586m; and TR14-Santa Cruz de la Zarza, with 5 009m. These two sites are quite similar, both in landscape composition (described further down) and in terms of the infrastructure associated with the railway line itself. Both areas were dominated by a flat running (less than 5m high) railway (41% of the total area for TR13 and 35% for TR14). The main difference between both areas, with regards to topography, is that the terrain in TR14 is slightly more ondulating, which translates into the railway infrastructure needing the presence of formation embankments (a raised platform to support the railway), constructed to avoid sudden changes in inclination of the railway (accounting 22% of the total area in TR14), whereas in TR13 there are no such embankments. With regards to features associated with the railway infrastructure, notable land features around the railway line include the proximity of (and intersection with) roads and the traditional railway (Fig 1). In TR13, the roads CM-301 and A-40 both form intersections under the HSR and the A-40 intersection features a 330m long viaduct for the HSR. In TR14 the roads A-40 and N-400 run parallel to the railway and the traditional railway (line 310 Aranjuez-Cuenca) intersects under the HSR, featuring a pergola on both sides of the intersection (not visible in Fig 1). Also, there is a railway maintenance station in TR13 (Fig 1, observable in the south side of the railway line in the eastern part of the census area). No permits were necessary in order to carry out the study because (i) all land accessed was public, (ii) there was no security concerns regarding the train traffic to be addressed because all observations were made from the outside of the security fence on both sides of the HSR infrastructure and (iii) all data was purely observational, not requiring the handling of any animals.

The study site is characterised by plains and open fields of dry land crops, most commonly cereal but also often legumes. The climate of the study site is continental Mediterranean (annual average temperature: 13.9°C, annual average precipitation: 437.8mm). Notably, in small sections to the north of the High-Speed railway, where the topography is more abrupt, natural vegetation can be found (holm oaks, pine trees, scrub, etc.). Parts of the study site, and nearby areas, are included as protected natural areas under different designations. A part of the Special Protection Area (SPA ES0000170) “Área esteparias de la Mancha Norte” can be found to the south of the study area. This protected denomination was awarded to the area due to its agricultural nature, which favours the presence of certain protected steppe species tied to this type of agricultural landscape. However, the limits of the SPA are not evidently linked to any changes in landscape, land use or topography in the study area, which is very homogenous; both within each census area and between them. This SPA is in itself included inside the Important Bird Area (IBA 193) “Tarancón-Ocaña-Corral de Almaguer”. The northernmost limit of the study area borders with the Site of Community Importance (SCI ES4250009) “Yesares del Valle del Tajo”. We conducted bird censuses, identifying them to the species level, when possible, using the eBIRD taxonomic criterion [42]. Bird censuses were carried out using two different methodologies: (i) linear transects and (ii) direct counting by car:

#### Field surveys

Linear transects were used to census smaller bird species. Species with a wingspan of less than 48cm, smaller than a red-legged partridge (*Alectoris rufa*), were considered small (from now on “Small Birds”). Each transect was 1 km in length and had no defined band width. Within each transect all birds detected were counted. Transects were evenly distributed in the 2 census areas, having each 2 sides of the HSR line. In each side of the HSR, transects were placed along 2 parallel lines, one by the rail and another 500m from the rail. There were 3 transects in each of these distance lines, thus producing a total of 24 transects, 12 in each census area (Fig 1). All transects were georeferenced and integrated in a Geographical Information System (GIS). In each transect an observer recorded all identifiable birds using, both by visual (using 10x42 binoculars) and hearing confirmation. For each observation; species, number of individuals, perpendicular distance to the center of the transect, time and date were recorded.

Direct counting by car was used in order to census large bird species in both census areas. Birds with a wingspan of more than 48cm were considered large (from now on “Large Birds”), and this methodology was used because their densities are too small to be accounted for with the linear transect methodology. Countings were performed along all possible service roads, in a band of 1km on each side of the car. The car moved at low speed (10—15km/h) and stopped at regular 500m intervals for 2–5 minutes. To avoid pseudorreplication among observers, after the census, data from all observers was contrasted to check for duplicated observations. The criteria for an observation to be considered duplicated was the following: (i) time passed between identical observations less than 30 minutes, (ii) similar number of individuals counted (we accepted a certain amount of error in bigger groups of birds), (iii) same direction of flight of the birds, (iv) proportion of sex and/or age ratio within a group of birds similar and (v) distance between the two observations was less than 500m. All observations were georreferenced using a Leica 1200 RF rangefinder and a Garmin 60CSx GPS. All data was displayed on a map using QGIS.

All censuses were carried out during the four hours after sunrise and the two hours before sunset (small variations possible in the face of changing weather conditions), in days in which there was good visibility and no rain. Days with hunting activity were avoided. Census were carried out in all study areas for two years, with sampling trips happening every season of the year (winter, spring, summer and autumn).

### Statistical analyses

Because of the differences in the methodology of field surveys between those carried out for Small Birds (Linear transects, see “Field Surveys”) and those carried out in order to census Large Birds (Direct Counting by Car), we separated the analysis of both datasets to avoid the emergence of confusion factors related to methodology. All statistical analyses were performed in R version 4.0.2 [43].

#### Small Birds statistical analysis

In the case of Small Birds, accounting for two years of surveys, we used 14566 observations belonging to 46 species. Due to the presence of big flocks of gregarious species (namely spotless starling, *Sturnus unicolor*), there was a large variation in the number of individuals observed between the first and second year of surveys, with 4761 individuals in the first year and 9805 in the second.

We estimated the effect of the presence of the HSR infrastructure on the number of observed birds by fitting a univariate mixed-effect model (Eq 1) using number of birds per transect and species as the response variable. The model included as fixed effects distance from the railway as a categorical variable with two levels (Railway, 500m; β_rail distance_), and an interaction term (β_season:year_) between season with four levels (Winter, Spring, Summer and Autumn; (β_season_) and year with two levels (first, second; β_year_). Selection of the fixed effects included in the model was done by an AIC criterion model selection analysis. Models that had ΔAIC < 4 between each other were inspected individually, to assess the best fit for the data. We controlled for the differences between the areas and units of survey in the study area by including transect as a random effect (b_k_). Furthermore, to control for the differences that different behaviours among species may have, we used species name (code-named Latin name or Latin_N) as an additional random effect (a_j_). We assumed a poisson distribution for the number of individuals observed. We performed a post-hoc analysis after the linear regression to obtain pairwise comparisons of the season, and year:season interaction terms of the model. We computed the marginal and conditional r-squared coefficients using Nakagawa’s R^2^ function [44].

$$\begin{array}{cc} \eta_{i}\sim & \text{Poisson}(\lambda_{i})\\ \text{log}(\lambda_{i})= & \alpha_{j[i],k[i]}+\beta_{\text{rail}\;\text{distance}}+\beta_{\text{season}}+\beta_{\text{year}}+\beta_{\text{season}:\text{year}}+a_{j}+b_{k}+\varepsilon_{j[i],k[i]}\\ a_{j}\sim & N\left(0,\sigma_{a_{j}}^{2}\right),\;\text{for}\;\text{latin\_N}\;\text{j}=1,\ldots ,\text{J}\\ b_{k}\sim & N\left(0,\sigma_{b_{k}}^{2}\right)\text{,}\;\text{for}\;\text{transect}\;\text{k}=1,\ldots ,\text{K} \end{array}$$ (1)

In order to evaluate whether the presence of the infrastructure affects the composition of the bird communities around it we, (i) performed a constrained ordination analysis using the Vegan package [45] and (ii) we analysed the bird community similarity between census sites, both close and far from the HSR infrastructure (see below). We performed a Detrended Correspondence Analysis (DCA) first to evaluate the length of the gradients, and evaluate the heterogeneity of the data (β diversity). We followed this with a redundancy analysis (RDA), as recommended by the observed DCA gradient lengths. Due to the gregarious behaviour of several species present within the data, we log transformed the response variable (number of observed birds) with the formula x’ = log(x + 1) in order to control for the effect that sporadic big flocks of birds have on the results. Year, season, and distance to rail were included as environmental variables for the RDA constrained analysis. To assess the significance of the environmental variables, we performed an ANOVA like permutation test by term with 1000 permutations, and considered the variance contained in an environmental variable to be statistically significant when the p-value was below 0.05. Additionally, in order to explore whether the presence of the infrastructure could affect the bird community in terms of habitat preference based on specific species traits, we used data from the AVONET project dataset [46] to group species in guilds determined by the major ecosystem in which the species occurs. With this data we constructed an alluvial plot that shows the frequency of observation of each guild in the transects by the rail and the transects 500 meters from the rail. We used this information to guide and support conclusions of the constrained ordination analyses.

Finally, in order to assess the homogenisation effects associated to the HSR infrastructure, we calculated a pairwise dissimilarity matrix using the species present and absent in the transects of different distance to the rail but in the same geographical location (same census area, TR13-Villarrubia de Santiago or TR14-Santa Cruz de la Zarza) and transects of the same distance to the rail, but in different locations. In order to do this we used the Jaccard dissimilarity index provided by the betapart package in R [47].

#### Large Birds statistical analysis

In the case of car surveys, because there was no survey unit (transect, point stations… etc.), each observation was georreferenced (see Field Surveys). To make use of this information we represented each observation over a map using QGIS (v 3.18.3). We then calculated the distance of each point observation to the railway infrastructure. This allowed to create a continuous distance variable, which was used for the linear modelling statistical analysis. For the subsequent constrained ordination analysis, we classified the distance variable into a two level factor variable, containing a 500m level and a Rail level. The factor variable was necessary to run the analysis, and the choice of a two level factor allowed to maintain consistency with the linear transect survey data.

Accounting for two years of surveys, we collected a total of 1548 observations of individuals belonging to 25 different species. Two species (*Columba livia* and *Columba palumbus*) had to be filtered out of the analysis due to them being included inconsistently in both survey methods, even though they both had wingspans of more than 48cm. Statistical analysis of this data followed the same pattern as for the linear transects data. We first modelled the effect of the presence of the HSR infrastructure on the number of observed birds per location and species with a univariate mixed-effect model (Eq 2). Fixed effects included in the model were distance to rail (as a continuous variable; β_rail distance_), and an interaction (β_season:year_) between season (factor term with four levels: Winter, Spring, Summer and Autumn; β_season_) and year of survey (factor term with two levels: first and second; β_year_). We included species name (a_j_) and location (d_l_) as random intercepts to control for the differences among survey sites and the variability among different species. We assumed a poisson distribution for the number of individuals observed.

$$\begin{array}{cc} \eta_{i}\sim & \text{Poisson}(\lambda_{i})\\ \text{log}(\lambda_{i})= & \alpha_{j[i],k[i]}+\beta_{\text{rail}\;\text{distance}}+\beta_{\text{season}}+\beta_{\text{year}}+\beta_{\text{season}:\text{year}}+a_{j}+b_{l}+\varepsilon_{j[i],l[i]}\\ a_{j}\sim & N\left(0,\sigma_{a_{j}}^{2}\right),\;\text{for}\;\text{latin\_N}\;\text{j}=1,\ldots ,\text{J}\\ b_{l}\sim & N\left(0,\sigma_{b_{l}}^{2}\right)\text{,}\;\text{for}\;\text{location}\;\text{l}=1,\ldots ,\text{L} \end{array}$$ (2)

Finally, following the same methodology as for the linear transect data, we performed a constrained ordination analysis and constructed an alluvial plot using species grouped into habitat guilds. We performed a DCA first and evaluated the gradient lengths. We followed this with a Canonical Correspondence Analysis (CCA). In order to avoid very common and gregarious birds masking the presence of rarer ones, we log transformed the observations of the individuals. Year, season and distance to rail (as a two level factor variable) were included as environmental variables for the CCA analysis. We followed the same methods as with the linear transect data in order to assess the statistical significance of the environmental variables.

## Results

### Small Birds

As previously mentioned, we collected observations for a total of 14566 individuals belonging to 46 different species. The most common species was the spotless starling (*Sturnus unicolor*) with 5149 observations. 6 species had only one observation. The mean number of individuals observed per transect in each year and season is 75.86 (82.27 SD). The mean number of individuals of each species observed per transect in each season was 11.53 (29.91 SD).

#### Effect of railway on number of individuals

**Fig 2:**
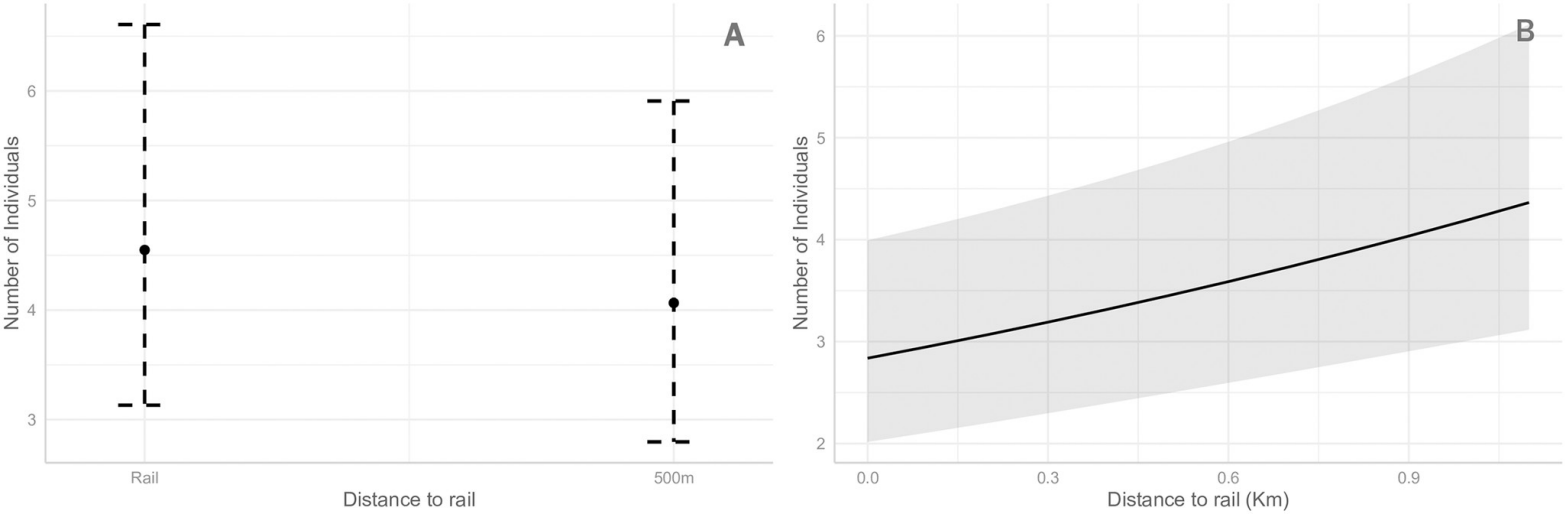
Variation in the number of birds observed in relation to the distance to rail. **A**. Estimated mean number of Small Bird individuals observed in relation to the distance to the railway. A two level factor was considered for the distance to rail: Rail (area immediately adjancent to the HSR infrastructure) and 500m (area 500m away from the HSR infrastructure). Vertical lines indicate 95% confidence intervals. **B**. Estimated mean number of Large Bird individuals observed in relation to the distance to the railway. A continuous scale of distance to rail expressed in km was considered in this case. Grey shaded area represents 95% confidence intervals.

**Fig 3:**
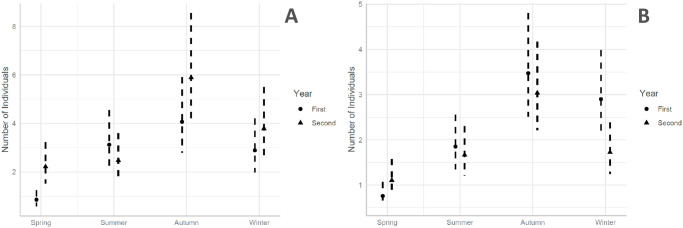
Variation in the number of birds observed in relation to season and year. **A**. Estimated mean numer of Small Bird individuals observed in relation to season (Autumn, Spring, Summer and Winter) and year of surveys (First: 2014–2015, Second: 2018–2019). The vertical lines represent 95% confidence intervals. **B**. Estimated mean number of Large Bird individuals observed in relation to season (Autumn, Spring, Summer and Winter) and year of surveys (First: 2014–2015, Second: 2018–2019). The vertical lines represent 95% confidence intervals.

Two models were closely contested in the model selection analysis (S1 Table). We decided against model averaging because, upon close inspection, estimates from both models only differed in the presence or absence of distance to rail. Thus, we kept the model that included distance to rail because it was our main variable of interest. In this model, mixed effects accounted for 84% of variation in the number of individuals ($R_{conditional}^{2}$; S1 Table), with fixed effects accounting for 17% of this variation ($R_{marginal}^{2}$; S1 Table). Change in the number of individuals with respect to the distance to the rail was not supported (Table 1, Fig 2A). The predicted number of birds in the rail area was 4.6 [95% CI 3.13, 6.61] and 4.07 [95% CI 2.80, 5.91] in the 500m area. However, there was a strong effect of the interaction between season and year with respect to the number of observed individuals (Table 1, Fig 3A), in which there was a significant decline in the number of observed individuals in Spring (Fig 3A). Finally, we found an effect of year of survey in the number of observed individuals (Table 1). The year effect is mainly driven by the difference found in Spring (Fig 3A). The predicted number of birds in the Spring of the first year was 0.62 [95% CI 0.42, 0.94], whereas it was 1.97 [95% CI 1.32, 2.95] for Spring of the second year (Fig 3A).

**Table 1:** Predictors of effect of different factors on number of birds per transect and species.

	Small Birds		Large Birds	
Fixed effects β	Estimate	95% CI	Estimate	95% CI
Intercept	1.40	[1.03, 1.78]*	1.04	[0.70, 1.38]*
Rail	0.11	[-0.14,0.36]	0.39	[0.21,0.57]*
Winter	-0.34	[-0.41, -0.27]*	-0.18	[-0.39, 0.02]
Spring	-1.56	[-1.65, -1.47]*	-1.52	[-1.76, -1.29]*
Summer	-0.26	[-0.35, -0.18]*	-0.63	[-0.83, -0.43]*
Second Year	0.37	[0.31, 0.43]*	-0.14	[-0.32 0.05]
Winter:Second Year	-0.10	[-0.19, -0.01]*	-0.39	[-0.66, -0.11]*
Spring:Second Year	0.58	[0.48, 0.69]*	0.51	[0.18, 0.84]*
Summer:Second Year	-0.60	[-0.71, 0.50]	0.03	[-0.23, 0.29]

Effect of different factors on the total number of individuals observed in the areas surrounding the HSR in a section of the Madrid—Castilla La Mancha—Comunidad Valenciana line in Toledo, Central Spain. Results shown belong to two different generalised linear mixed regression models, which are divided between Small Birds and Large Birds (See Statistical Analyses for further detail).

#### Effect of railways on bird communities

**Fig 4:**
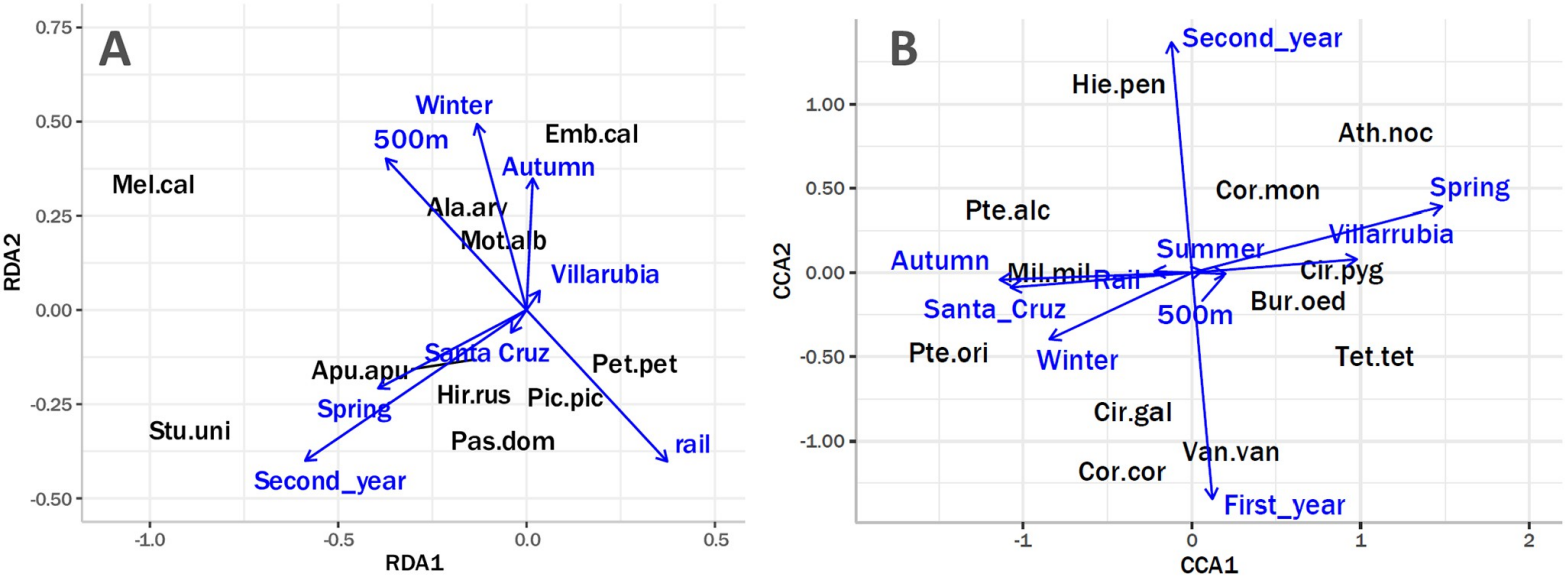
Multivariate analysis plots of enviromental factors in relation to different bird species. **A**. Ordination diagram of Redundancy Analysis (RDA). The most common species of Small Birds detected in the areas of survey are represented, along with blue arrows representing the environmental variables. The length of the arrows represents the relative weight of the variable in explaining the variance in the bird community. Species names are abbreviated by showing the first three letters of the genus and specific names separated by a point. **B**. Ordination diagram of Canonical Correspondence Analysis (CCA). The most common species of Large Birds detected in the areas of survey are represented, along with blue arrows representing the environmental variables. The length of the arrows represents the relative weight of the variable in explaining the variance in the bird community. Species names are abbreviated by showing the first three letters of the genus and specific names separated by a point.

In the DCA exploratory analysis, we observed that the spatial variability of the bird communities around the HSR infrastructure was medium, with a maximum gradient length of 3.06. Proceeding with the RDA analysis, we concluded that the environmental variables could explain 21.8% of the variability in the sample. Axes RDA1 and RDA2 (represented in Fig 4A) amounted to a 68% of the constrained inertia. All environmental factors included in the analysis had a significant effect on bird communities surrounding the HSR infrastructure (Table 2). Specifically, the distance from the HSR infrastructure had a significant effect on the community of birds (Table 2, Fig 4A). The effect of distance to rail affected several species and explained the same amount of variation as season (Table 2, Fig 4A). For instance, *Melanocorypha calandra* is strongly positioned away from the railway, showing a very strong rejection of the railway infrastructure (Fig 4A). On the contrary, species like *Petronia petronia, Passer domesticus* or *Pica pica* show the opposite tendency, albeit more moderate (Fig 4A). Furthermore, several species, such as *Alauda arvensis, Hirundo rustica* and *Apus apus* are positioned near certain seasons of the year (Fig 4A), evidencing changes in the presence of these species throughout the year. Finally, and as previously explained, there is a strong effect of year, mainly explained by the species *Sturnus unicolor* and *Passer domesticus*, both of gregarious nature (Table 2, Fig 4A). Finally, in the alluvial plot (S1A Fig) we observed that from our species, the species that occur mainly in grassland habitats had slightly higher frenquencies 500 meters from the rail. Also, species that occur mainly in human modified, woodland and especially rock habitats, had a tendency to be spotted by the rail with higher frenquency (S1A Fig).

**Table 2:** Variance in the community of birds surrounding the HSR explained by different environmental variables.

	Small Birds			Large Birds		
Environmental variables	Variance	F	p-value	Variance	F	p-value
Season	0.11	4.82	<0.001*	0.38	1.75	<0.001*
Distance to Rail	0.09	10.82	<0.001*	0.07	1.00	0.46
Year	0.16	17.03	<0.001*	0.14	1.88	0.006*
Location	0.02	2.87	0.012*	0.20	2.71	<0.001*
Season:Distance to Rail	0.05	1.89	0.010*	0.20	0.91	0.69
Residual	1.45			3.90		

Results shown belong to two different constrained ordination analyses. The first one, a Redundancy Analysis (RDA) was done for Small Birds. The second one, a Canonical Correlation Analysis (CCA), was done for Large Birds (See Statistical Analyses for further detail).

**Fig 5:**
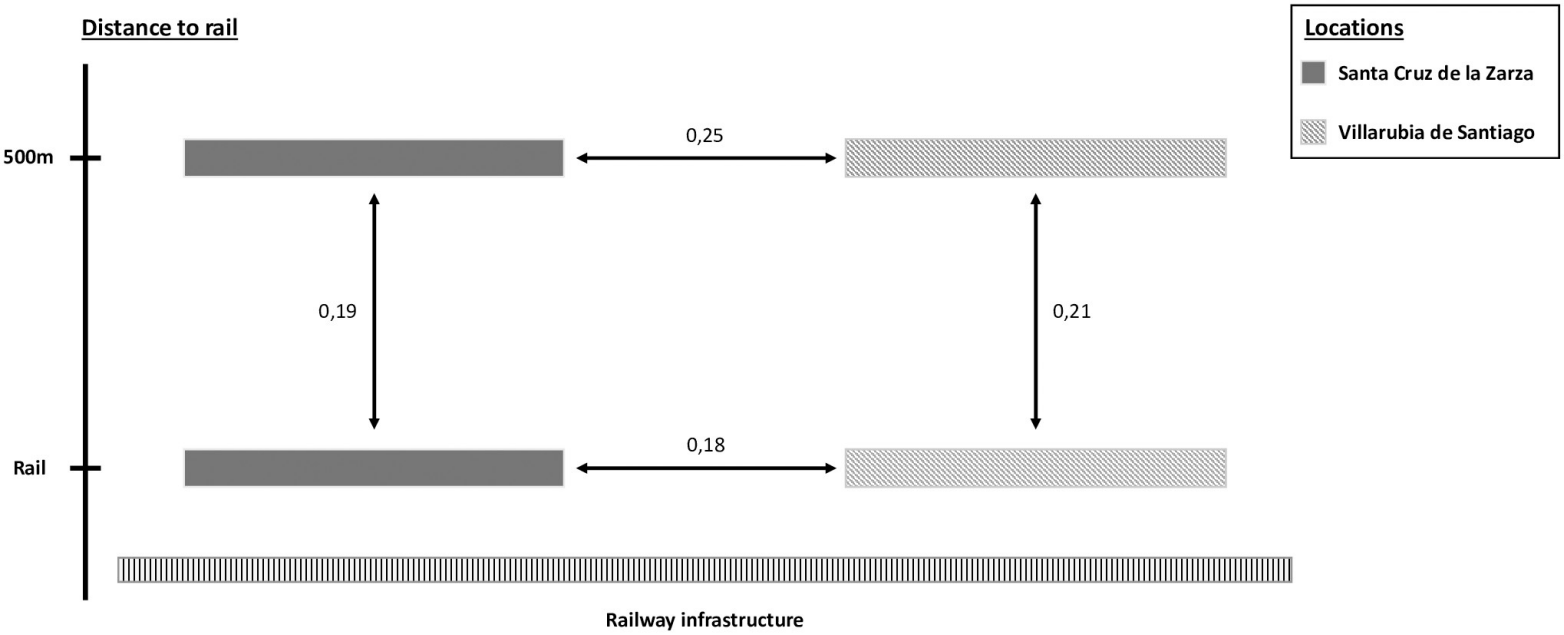
Diagram of pairwise dissimilarity matrix for Small Birds. Diagram expresses the difference in species composition between different areas of survey. The railway infrastructure is represented at the bottom. Transects belonging to the areas of surveys (Santa Cruz de la Zarza and Villarubia de Santiago) are represented using two different colours. The vertical axis represents distance to the rail (Rail and 500m). Numbers show the results given by the Jaccard dissimilarity index, expressing how different are the communities in each of the areas compared.

These community changes caused by the presence of the rail were further confirmed by the Jaccard Dissimilarity Index. We observed that the dissimilarity between the transects near the rail infrastructure and 500m away from it in Villarubia de Santiago was of 0.21 (Fig 5). In the case of Santa Cruz de la Zarza, this dissimilarity was of 0.19. However, for transects close to the rail but on different locations, the dissimilarity amounted to 0.18; and it was 0.25 for transects 500m away from the rail belonging to different locations (Fig 5). Thus, the lowest dissimilarity was found when comparing transects near the rail from different locations and the highest when comparing transects far away from the rail in different locations. These dissimilarity indexes evidence changes in the community composition correlated to the distance to the rail.

### Large Birds

In the case of direct counting by car, we collected observations for a total of 1609 individuals belonging to 28 different species of medium to Large Birds. In this case the most common species was *Otis tarda* with 744 observations and 2 species had just one observation. The mean number of observations per year, location and season was 100.56 (56.35 SD). The mean for the number of individuals of each species observed per location, in each year and season was 10.80 (23.55 SD). Note that in the case of Large Birds there were no transects and so these means are for the total counts in each location surveyed.

#### Effect of railway on number of individuals

There were two models in the model selection that shared 98% of the weight of explanatory power (S2 Table). However the second model (which included an interaction between distance to rail and season) had convergence issues. Thus, we decided against model averaging and used the top model (which included distance to rail and an interaction between year and season; S2 Table). The mixed effect model accounted for 59% of variation ($R_{conditional}^{2}$, S2 Table), of which 23% was explained by the fixed effects ($R_{marginal}^{2}$, S2 Table). An effect of rail distance on the number of birds observed was strongly supported (Table 1). We observed an increase in the number of birds as the distance to the rail increases (Fig 2B). The effect of season was significant for Spring and Summer when using Autumn as the intercept (Table 1). In the post-hoc contrasts, all comparisons between season were significant except for Winter-Autumn (S4 Table, Fig 3B). Furthermore, both an increase in the number of birds in the Spring of the second year and a decrease in the Winter of the second year were supported by the model (Table 1, Fig 3B) when using Autumn as intercept, whereas all post-hoc comparisons between first year and second year in each season were significant (S4 Table, Fig 3B).

#### Effect of railways on bird communities

In the exploratory DCA analysis, we found that the spatial variability of the larger birds around the HSR infrastructure was medium to high, with a maximum gradient length of 4.96. Thus, we proceeded with the CCA analysis which concluded that the environmental variables could explain 21.1% of the total inertia of the model. Axes CCA1 and CCA2 (represented in Fig 4B) expressed 58% of the constrained inertia. Among the environmental factors, season, year and location had a significant effect on the communities around the HSR infrastructure (Table 2). In Fig 4B it can be observed that the effect of distance to rail is relatively small, compared to the rest of environmental variables. On the other hand, we observed that season has the largest effect on the variance distribution, with species like *Tetrax tetrax, Circus pygargus, Athene noctua* and *Burhinus oedicnemus* more commonly detected in Spring. Species such as *Pterocles orientalis, Vanellus vanellus* and *Milvus milvus* were more commonly associated with the Autumn and Winter season. *Columba Palumbus*, *Streptopelia decaocto* or *Circaetus gallicus* were more commonly observed during the Summer. Furthermore, another environmental variable that explained a high proportion of the constrained variability was location. On one hand, *Pterocles alchata* was clearly associated with the location of TR14-Santa Cruz de la Zarza, whereas on the other hand species like *Athene noctua* was associated with TR13-Villarubia de Santiago. The variable year, even though significant, is observed to have a small effect in Fig 4B and no species are observed to be specifically associated with it. In the alluvial plot, representing the habitat guilds for the large bird species (S1B Fig), we observed that shrubland and forest species had slightly higher observation frequencies near the railway, compared to 500m. Grassland and human modified showed an even smaller tendency in the opposite direction (S1B Fig).

## Discussion

Understanding how the presence of infrastructure affects the bird communities around it is of vital importance to direct conservation and mitigation measures as high-speed railways develop, since species attracted to the HSR face a higher risk of collision with trains, whereas species rejecting the area may suffer from landscape fragmentation. Our results show that the presence of HSR infrastructure has a species-specific effect on the bird communities around it, producing some species substitution close to the infrastructure associated with (i) the rejection of the HSR by some species combined with (ii) a higher frequency of others near the rail in comparison with the wider landscape. As a final result, (iii) a process of species filtering takes place leading to a local biotic homogenisation along the HSR that hinders the natural species turnover irrespective of landscape changes at kilometric scales.

The total number of birds surrounding the HSR infrastructure was not greatly affected by the presence of the infrastructure itself. For smaller bird species, there was no significant change in the number of total observed individuals between areas directly adjacent to the railway and areas 500m away from it (Table 1; Fig 2A). Past studies generally show a decline in the number of individuals and species in the areas near roads carrying frequent traffic ([48–50]; see [26] for a review). In the case of railways, studies have generally found that there is no effect in the number of individuals, or even positive effects [10, 21, 22, 38]. Our results, when looking at total number of individuals, are in line with past studies done in railways. In the case of larger bird species, however, we found that as the distance to the rail increases so does the number of individuals observed, although the magnitude of the effect was small (Table 1; Fig 2A). Thus, our results show that, looking at general numbers of individuals, there is a small rejection of the infrastructure by larger bird species, and no effect on smaller species. Although the rejection of the infrastructure by larger birds contradicts the results found by [21], the size of the birds and the species surveyed in this instance could have an effect on the behaviour of the birds. [21] showed that mostly insectivores were attracted to the rail infrastructure, none of which are large species of birds.

One of the main reasons why birds are thought to avoid the vicinity of busy roads is noise disruption caused by passing cars [49, 51–53]. Noise pollution in railways is reduced due to the intermittent nature of railroad traffic. Furthermore, noise pollution in HSRs may be further reduced due to the straighter tracks required for high traveling speeds, which reduces the slipping of the metal wheels and thus noise [54]. Studies that have found railways to have a positive effect on the number of individuals in the area near the infrastructure have found that it’s mainly due to the niches that these infrastructures open for several bird species [10, 21, 34, 38]. For example, the catenary associated with the railway infrastructure makes for great perching sites; and viaducts and other areas near the railway are frequently used as nesting sites [10]. However, a study done on meadow birds correlated a reduction in avian density with railway noise pollution [55]. These differing results point out the fact that effects of presence of the railway may be multifactorial, being species specific and landscape specific. Our results point towards this possibility, showing that the response of the avian communities near HSR infrastructure may differ depending on several different factors.

Intra- and interannual variation, however, did affect the general number of individuals. More individuals were observed in the autumn (Table 1; Fig 3A and 3B), with post-hoc contrasts showing significant differences between all seasons except for the summer-winter contrast for Small Birds, and the autumn-winter contrast for Large Birds. These differences in abundance between seasons are to be expected, due to the migratory nature of several species present in the study area [56]. The general trend of higher number of individuals during autumn and winter, compared to the number of individuals in spring and summer (Fig 3A and 3B) could be related to a change in food availability in this agricultural study area [57]. The number of individuals observed also varied across years of survey (Table 1; Fig 3A and 3B). This difference across years varies depending on the season of the year (Small Birds: S3 Table; Fig 3A; Large Birds: S4 Table; Fig 3B). Notably, in Fig 4A it can be observed that the spotless starling (*Sturnus unicolor*), a gregarious species, is strongly associated with the environmental variables “Spring” and “Second Year”. These differences are driven by the presence of gregarious species which can be observed in groups of several hundred individuals, combined with the stochasticity inherent to the study of wild populations which has the effects of large groups of birds being occasionally spotted throughout the surveys.

Instead of a change in the general number of birds, what we observed in the bird community surrounding the HSR was a species substitution effect, with the distance to the rail variable having a similar magnitude to that of season (Table 2). Our results show that, for certain species, the presence of the high-speed rail infrastructure is comparable to the seasonal changes in species composition affecting migratory species. Observing the results shown in Table 2 and Fig 4A, the species specific effect of the presence of the rail infrastructure becomes evident for smaller birds. For example, on one hand, *Melancorypha calandra* was strongly associated with the areas 500m from the rail, and rarely observed in the areas near the rail (Fig 4A). On the other hand, species such as *Hirundo rustica, Passer domesticus, Pica pica* and *Petronia petronia* were observed with higher frequencies in the area near the HSR infrastructure (Fig 4A). This result is supported by the alluvial plot (S1 Fig) which shows that species that are tied to grassland habitats tend to avoid the vicinity of the railway (*Melancorypha calandra*), while species that are tied to human modified (*Hirundo rustica, Passer domesticus, Pica pica*) or rock habitats *Petronia petronia* tend to prefer the vicinity of the railway. However, more research is necessary to quantitatively assess whether the railway may be selectively attracting or rejecting species based on habitat preference. For the larger bird species, we found that there was no species that was specifically associated with the railway or the areas away from it (Table 2), and in Fig 4B it can be observed that the arrows representing the variability explained by distance to rail are very short and no species are associated with them. This is further shown by the alluvial plot, which shows very small tendencies for either distance category (S1 Fig). For larger species of birds, generally, the whole community seems to reject the railway area (Table 1; Fig 2B), rather than just certain species. However, it is important to note that the observed effect, whilst significant, it’s not large. Past studies show that raptors in general, hunt along transport lines, but insectivores and granivorous species have been observed to forage near transport infrastructure as well [21, 53, 58]. These studies, and others that show both the negative and positive effects of transport lines on bird populations surrounding it [10, 18, 34, 38], highlight the complex nature of the relationship bird communities have with transport infrastructures.

Our results on the dissimilarity matrix provide some further insight on the species-specific nature of the effects of the presence of the HSR infrastructure on bird communities, highlighting the potential of this effect to be carried across landscape changes (Fig 5). We found that dissimilarity was at its maximum in the areas that were 500m from the railway but in different study sites; and at its minimum in the areas near the railway but in different sites. These results suggest that the presence of HSR infrastructure may cause biotic homogenisation in the areas near it. It is possible that this is due to the reduction of the natural turnover of species as the railway extends over the territory, as it attracts the same species regardless of changes in the general landscape, and filters out others. In a study carried out in Madrid (Central Spain), [52] showed that bird abundance increased in urban areas due to the higher presence of urban-exploiter species, and that roads in between had a buffer area on either side that caused a decrease of the native avifauna. This study, in combination with the positive effects of transport lines on avian communities previously mentioned [10, 21] and our own results, support the hypothesis of HSR infrastructure causing biotic homogenisation due to the effect it has on the species composition of the communities that surround it. However, it is important to note that there were only two survey locations and that they were not very distant from each other, as they were separated by 15km. This may limit our inference power in this regard, since the landscape changes possible in this distance are not as notable as they might be if the distance between areas was larger and the number of census locations higher. However, the effect of the presence of the railway is still discernable at short distances, showing that the railway may have homogenising effect in the community that surrounds it. Further investigation of how landscape changes in species composition might be muffled by the homogenising effect of the presence of HSR infrastructure would shed further light on the large scale effects of human-made infrastructures on bird communities around them.

## Conclusion

In conclusion, our results show that the effects of the presence of the HSR are notable and significant. We show that these effects can be discerned from, and are comparable to the effects seasonal changes have in the species composition in bird communities around the HSR infrastructure. Furthermore, we show that the presence of the HSR infrastructure can affect the natural species turnover as the landscape changes, although further research on this aspect is necessary. Our results shed light on how the operation of current HSR lines and their future development can affect communities around them. We propose that species-specific and habitat modification focused research is necessary to assess the risk bird communities are exposed to by the development of high-speed railways, and in order to develop conservation and mitigation measures to protect vulnerable species.

## Supporting information

S1 TableModel selection output for the Small birds model.Predictors: a summary of fixed effects and interactions taken into account; k: number of parameters modelled; w: akaike information criterion (AIC) weight; Δ*AIC*: difference in akaike information criterion (AIC) between any given model and the top model. Top model had an AIC value of 18760.2; $R_{conditional}^{2}$: proportion of variance explained by mixed effects; $R_{marginal}^{2}$: proportion of variance explained by fixed effects.(PDF)

S2 TableModel selection output for the Large Birds model.Predictors: a summary of fixed effects and interactions taken into account; k: number of parameters modelled; w: akaike information criterion (AIC) weight; Δ*AIC*: difference in akaike information criterion (AIC) between any given model and the top model. Top model had an AIC value of 18760.2; $R_{conditional}^{2}$: proportion of variance explained by mixed effects; $R_{marginal}^{2}$: proportion of variance explained by fixed effects.(PDF)

S3 Table Post-Hoc pairwise comparison of the interaction terms for the chosen model for Small Birds. Contrasts between seasons and between years during the same season are shown.
(PDF)

S4 Table Post-Hoc pairwise comparison of the interaction terms for the chosen model for Large Birds. Contrasts between seasons and between years during the same season are shown.
(PDF)

S5 Table Summary species table. Bird community composition in relation to the distance to the HSR infrastructure (500m from the rail and rail) in Central Spain. Communities shown are separated between Small Birds and Large Birds (see Methods for more details).
(PDF)

S1 Fig Alluvial plots of habitat preference guilds in relation to distance to rail. Relationship between the frequency of observation of species classified in guilds by the major ecosystem in which the species occurs (based on Avonet information of bird species) and the distance to rail. Panel **A** represents small bird species classified by main Habitat Preference, and panel **B** represents large bird species classified by main Habitat Preference.
(PDF)
