# Supplementary material for: High-speed railway infrastructure leads to species-specific changes and biotic homogenisation in surrounding bird community
Source: PLoS One. 2024 Apr 10;19(4):e0301899. doi: 10.1371/journal.pone.0301899 (PMC11006141; doi:10.1371/journal.pone.0301899)
Supplement: S1 Table — Predictors: a summary of fixed effects and interactions taken into account; k: number of parameters modelled; w: akaike information criterion (AIC) weight; ΔAIC: difference in akaike information criterion (AIC) between any given model and the top model. Top model had an AIC value of 18760.2; Rconditional2: proportion of variance explained by mixed effects; Rmarginal2: proportion of variance explained by fixed effects. (PDF) [file pone.0301899.s001.pdf]

Table S1: **Model selection output for the Small birds Model.** Predictors: a summary of fixed effects and interactions taken into account; k: number of parameters modelled; w: akaike information criterion (AIC) weight; ΔAIC: difference in akaike information criterion (AIC) between any given model and the top model. Top model had an AIC value of 18760.2; $R^2_{conditional}$: proportion of variance explained by mixed effects; $R^2_{marginal}$: proportion of variance explained by fixed effects.

**Predictors**	k	w	ΔAIC	$R^2_{conditional}$	$R^2_{marginal}$
Season * year	10	0.65	0.00	0.84	0.17
Distance to rail + season * year	11	0.35	1.28	0.84	0.17
Distance to rail * season + year	11	0.00	302.71	0.83	0.13
Distance to rail * year + season	9	0.00	344.83	0.83	0.13
Season + year	7	0.00	357.81	0.83	0.13
Distance to rail + season + year	8	0.00	358.96	0.83	0.13
Ditance to rail * season	10	0.00	662.74	0.83	0.12
Season	6	0.00	744.05	0.83	0.12
Distance to rail + season	7	0.00	745.44	0.83	0.12
Distance to rail * year	6	0.00	2781.72	0.82	0.04
Year	4	0.00	2838.02	0.82	0.03
Distance to rail + year	5	0.00	2838.94	0.82	0.03
Null	3	0.00	3421.36	0.82	0.00
Distance to rail	4	0.00	3422.39	0.82	0.00
