# Supplementary material for: High-speed railway infrastructure leads to species-specific changes and biotic homogenisation in surrounding bird community
Source: PLoS One. 2024 Apr 10;19(4):e0301899. doi: 10.1371/journal.pone.0301899 (PMC11006141; doi:10.1371/journal.pone.0301899)
Supplement: S2 Table — Predictors: a summary of fixed effects and interactions taken into account; k: number of parameters modelled; w: akaike information criterion (AIC) weight; ΔAIC: difference in akaike information criterion (AIC) between any given model and the top model. Top model had an AIC value of 18760.2; Rconditional2: proportion of variance explained by mixed effects; Rmarginal2: proportion of variance explained by fixed effects. (PDF) [file pone.0301899.s002.pdf]

Table S2: **Model selection output for the Large Birds model.** Predictors: a summary of fixed effects and interactions taken into account; k: number of parameters modelled; w: akaike information criterion (AIC) weight; ΔAIC: difference in akaike information criterion (AIC) between any given model and the top model. Top model had an AIC value of 18760.2; $R^2_{conditional}$: proportion of variance explained by mixed effects; $R^2_{marginal}$: proportion of variance explained by fixed effects.

**Predictors**	***k***	***w***	ΔAIC	$\mathbf{R^2_{conditional}}$	$\mathbf{R^2_{marginal}}$
Distance to rail + year*season	11	0.66	0.00	0.60	0.24
Distance to rail*season + year	11	0.33	1.38	0.60	0.23
Distance to rail*season	10	0.01	9.48	0.59	0.23
Year*season	10	0.00	15.45	0.61	0.23
Distance to rail + year + season	8	0.00	19.69	0.58	0.22
Distance to rail*year + season	9	0.00	21.68	0.58	0.22
Distance to rail + season	7	0.00	25.58	0.58	0.22
Season + year	7	0.00	34.27	0.59	0.22
Season	6	0.00	41.88	NA	0.34
Distance to rail	4	0.00	268.60	0.52	0.02
Distance to rail + year	5	0.00	270.49	0.52	0.02
Distance to rail*year	6	0.00	272.47	0.52	0.02
Null	3	0.00	290.52	0.51	0.00
Year	4	0.00	291.95	0.52	0.00
