# Supplementary material for: High-speed railway infrastructure leads to species-specific changes and biotic homogenisation in surrounding bird community
Source: PLoS One. 2024 Apr 10;19(4):e0301899. doi: 10.1371/journal.pone.0301899 (PMC11006141; doi:10.1371/journal.pone.0301899)
Supplement: S3 Table — Contrasts between seasons and between years during the same season are shown. (PDF) [file pone.0301899.s003.pdf]

Table S3: **Post-Hoc pairwise comparison of the interaction terms for the chosen model for Small Birds.** Contrasts between seasons and between years during the same season are shown.

Contrast	Estimate	Std. Error	Z value	p-value
Spring - Autumn	-1.56	0.05	-33.57	<0.001
Summer - Autumn	-0.26	0.04	-6.19	<0.001
Winter - Autumn	-0.34	0.04	-9.38	<0.001
Summer - Spring	1.30	0.05	24.86	<0.001
Winter - Spring	1.22	0.05	25.84	<0.001
Winter - Summer	-0.08	0.04	-1.76	0.29
Autumn,first - Autumn,second	-0.37	0.03	-12.19	<0.001
Spring,first - Spring,second	-0.95	0.05	-21.20	<0.001
Summer,first - Summer,second	0.23	0.05	5.12	<0.001
Winter,first - Winter,second	-0.27	0.03	-8.14	<0.001
