# Supplementary material for: High-speed railway infrastructure leads to species-specific changes and biotic homogenisation in surrounding bird community
Source: PLoS One. 2024 Apr 10;19(4):e0301899. doi: 10.1371/journal.pone.0301899 (PMC11006141; doi:10.1371/journal.pone.0301899)
Supplement: S4 Table — Contrasts between seasons and between years during the same season are shown. (PDF) [file pone.0301899.s004.pdf]

Table S4: **Post-Hoc pairwise comparison of the interaction terms for the chosen model for Large Birds.** Contrasts between seasons and between years during the same season are shown.

**Contrast**	**Estimate**	**Std. Error**	**Z value**	**p-value**
Spring - Autumn	-1.52	0.12	-12.71	<0.001
Summer - Autumn	-0.63	0.10	-6.30	<0.001
Winter - Autumn	-0.18	0.10	-1.73	0.31
Summer - Spring	0.89	0.12	7.76	<0.001
Winter - Spring	1.34	0.12	11.04	<0.001
Winter - Summer	0.45	0.10	4.41	<0.001
Autumn,first - Autumn,second	0.14	0.10	1.40	0.86
Spring,first - Spring,second	-0.38	0.14	-2.65	0.14
Summer,first - Summer,second	0.11	0.10	1.07	0.96
Winter,first - Winter,second	0.52	0.11	4.86	<0.001
