# Supplementary material for: High-speed railway infrastructure leads to species-specific changes and biotic homogenisation in surrounding bird community
Source: PLoS One. 2024 Apr 10;19(4):e0301899. doi: 10.1371/journal.pone.0301899 (PMC11006141; doi:10.1371/journal.pone.0301899)
Supplement: S5 Table — Bird community composition in relation to the distance to the HSR infrastructure (500m from the rail and rail) in Central Spain. Communities shown are separated between Small Birds and Large Birds (see Methods for more details). (PDF) [file pone.0301899.s005.pdf]

Table S5: **Summary species table.** Bird community composition in relation to the distance to the HSR infrastructure (500m from the rail and rail) in Central Spain. Communities shown are separated between Small Birds and Large Birds (see Methods for more details).

Small Birds				Large Birds			
**Species**	**500m**	**Rail**	**Habitat**	**Species**	**500m**	**Rail**	**Habitat**
Alauda arvensis	265	60	Grassland	Accipiter gentilis	1	1	Forest
Anthus pratensis	4	5	Grassland	Alectoris rufa	73	164	Shrubland
Apus apus	31	31	Human Modified	Athene noctua	11	2	Human Modified
Calandrella- -brachydactyla	20	3	Grassland	Burhinus oedicnemus	13	7	Grassland
Carduelis carduelis	462	1203	Woodland	Buteo buteo	37	35	Grassland
Cecropis daurica	0	9	Grassland	Circaetus gallicus	3	1	Woodland
Chloris chloris	11	31	Woodland	Circus aeruginosus	3	8	Wetland
Cisticola juncidis	1	0	Grassland	Circus pygargus	4	8	Grassland
Clamator- -glandarius	0	1	Shrubland	Corvus corax	3	3	Forest
Coturnix coturnix	2	0	Human Modified	Corvus corone	73	52	Human Modified
Curruca- -conspicillata	1	16	Shrubland	Corvus monedula	6	0	Human Modified
Curruca iberiae	1	3	Shrubland	Falco columbarius	1	1	Woodland
Curruca- -melanocephala	6	5	Shrubland	Falco peregrinus	0	1	Grassland
Cyanistes caeruleus	1	0	Forest	Gyps fulvus	2	3	Grassland
Emberiza calandra	510	761	Grassland	Hieraaetus pennatus	2	5	Forest
Fringilla coelebs	28	10	Forest	Milvus migrans	0	1	Woodland
Galerida cristata	429	602	Grassland	Milvus milvus	13	9	Shrubland
Hippolais polyglotta	1	1	Woodland	Otis tarda	504	240	Grassland
Hirundo rustica	22	30	Human Modified	Phalacrocorax carbo	0	2	Wetland
Lanius meridionalis	4	2	Shrubland	Pterocles alchata	19	19	Grassland
Lanius senator	1	6	Shrubland	Pterocles orientalis	37	5	Grassland
Linaria cannabina	304	685	Shrubland	Streptopelia decaocto	13	6	Human Modified
Melanocorypha- -calandra	1981	76	Grassland	Tetrax tetrax	44	21	Grassland
Merops apiaster	6	7	Woodland	Vanellus vanellus	17	1	Wetland
Motacilla alba	70	86	Human Modified				
Oenanthe hispanica	0	1	Shrubland				
Oenanthe oenanthe	4	13	Grassland				

Table S5 – continued from previous page

Small Birds				Large Birds			
**Species**	**500m**	**Rail**	**Habitat**	**Species**	**500m**	**Rail**	**Habitat**
Oriolus oriolus	1	0	Woodland				
Parus major	11	20	Woodland				
Passer domesticus	154	647	Human Modified				
Passer hispaniolensis	0	36	Shrubland				
Petronia petronia	19	210	Rock				
Phoenicurus- -ochruros	3	10	Rock				
Phylloscopus- -collybita	0	1	Forest				
Pica pica	121	189	Human Modified				
Pyrrhocorax- -pyrrhocorax	0	2	Rock				
Saxicola rubicola	2	5	Shrubland				
Serinus serinus	27	21	Forest				
Streptopelia- -decaocto	1	3	Human Modified				
Sturnus unicolor	2408	2741	Forest				
Sylvia atricapilla	0	3	Woodland				
Turdus merula	1	1	Forest				
Turdus philomelos	59	1	Forest				
Upupa epops	10	7	Grassland				
