# Supplementary material for: High-speed railway infrastructure leads to species-specific changes and biotic homogenisation in surrounding bird community
Source: PLoS One. 2024 Apr 10;19(4):e0301899. doi: 10.1371/journal.pone.0301899 (PMC11006141; doi:10.1371/journal.pone.0301899)
Supplement: S1 Fig — Relationship between the frequency of observation of species classified in guilds by the major ecosystem in which the species occurs (based on Avonet information of bird species) and the distance to rail. Panel A represents small bird species classified by main Habitat Preference, and panel B represents large bird species classified by main Habitat Preference. (PDF) [file pone.0301899.s006.pdf]

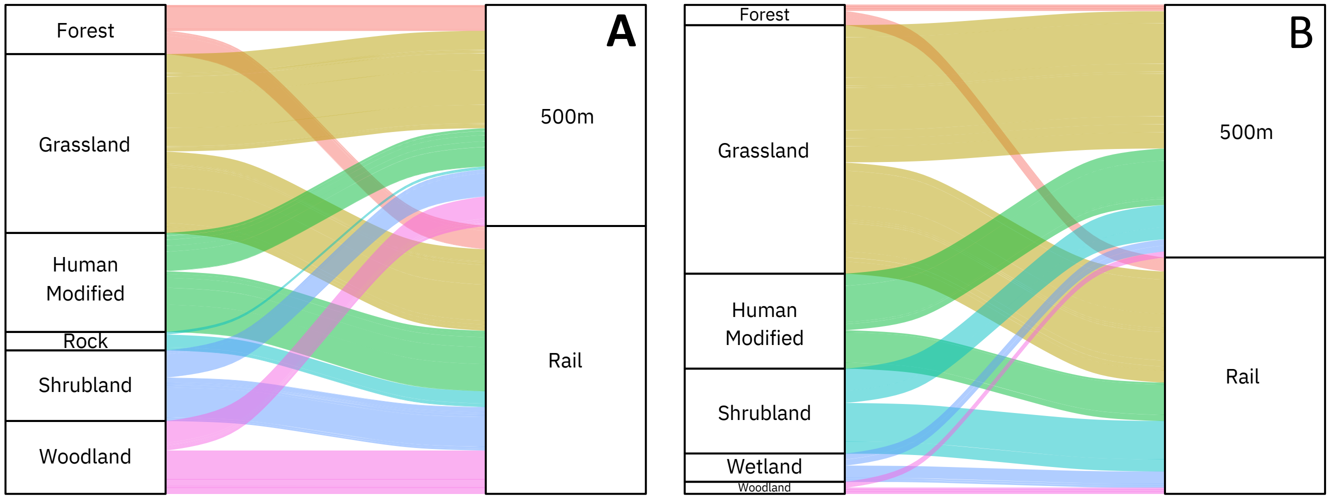

Figure S1: **Alluvial plots of Habitat Preference Guilds in relation to Distance to Rail.** Relationship between the frequency of observation of species classified in guilds by the major ecosystem in which the species occurs (based on Avonet information of bird species) and the distance to rail. Panel **A** represents small bird species classified by main Habitat Preference, and panel **B** represents large bird species classified by main Habitat Preference.
